# Imaging pathways in spondyloarthritis: integrating radiography, ultrasonography, magnetic resonance imaging, low-dose computed tomography, and artificial intelligence methods: Radiology and AI in SpA

**DOI:** 10.1007/s00296-025-06051-7

**Published:** 2025-12-15

**Authors:** Magdalena Z. Morytko, Patrycja S. Matusik, Renata Wawrzyniak, Tadeusz J. Popiela

**Affiliations:** 1 Student’s Scientific Group of Radiology, Jagiellonian University Medical College, 30-688 Kraków, Poland; 2 Department of Diagnostic Imaging, University Hospital, 30-688 Kraków, Poland; 3 Jagiellonian University Medical College, 30-688 Kraków, Poland; 4 Department of Radiology and Diagnostic Imaging, 5th Military Clinical Hospital with Polyclinic, Independent Public Health Care Institution, Kraków, Poland

**Keywords:** Artificial intelligence, Computed tomography, Deep learning, Magnetic resonance imaging, Sacroiliac joint, Spondyloarthritis

## Abstract

Spondyloarthritis (SpA) refers to a family of chronic inflammatory rheumatic conditions characterized by axial and/or peripheral manifestations. Early detection of SpA is crucial for improving long-term patient outcomes and necessitates a refined diagnostic algorithm. This literature review addresses current recommendations for imaging approaches in SpA, proposes a contemporary diagnostic algorithm for suspected axial SpA, and discusses current and emerging applications of artificial intelligence (AI) in diagnosis and management. A comprehensive literature search of PubMed, Embase and Scopus was performed for studies published between January 2010 and August 2025. Relevant English-language studies on imaging modalities and AI applications in SpA were included after independent screening. The implementation of advanced imaging techniques—such as low-dose computed tomography (CT) for detailed structural assessment and standardized magnetic resonance imaging (MRI) protocols for detecting inflammatory changes—has improved the diagnostic evaluation of sacroiliac joints. Incorporating clinical features and modality-specific strengths helps tailor imaging choices to individual patients with suspected SpA. In clinical practice, MRI may be considered for early detection of sacroiliitis—especially in younger patients and those with short symptom duration—whereas conventional radiography continues to serve as the recommended first-line imaging modality in many diagnostic pathways. Low-dose CT should be reserved for selected cases, such as inconclusive MRI findings, contraindications to MRI, limited MRI availability, or a specific need to assess structural damage. Advances in AI, particularly in deep learning, have had a remarkable impact on medical research. Despite existing limitations, such as costs of deployment and medico-legal considerations, their role in rheumatological imaging is being actively investigated. Deep learning-based models trained on radiographic, CT and MRI datasets have demonstrated progressively greater precision in detecting sacroiliitis, becoming a powerful tool that complements human judgement. Prospective strategies integrating multimodal imaging, AI-assisted interpretation, and prognostic assessment may enhance diagnostic accuracy and provide personalized therapeutic solutions in SpA.

## Introduction

Spondyloarthritis (SpA) is a group of chronic inflammatory rheumatic disorders that share common clinical features, pathogenic mechanisms, and genetic predisposition [1, 2]. In addition to musculoskeletal involvement, patients may also present with extra-articular manifestations such as psoriasis, inflammatory bowel disease (IBD), and acute anterior uveitis [3]. Early and accurate assessment of SpA remains challenging due to the scarcity of clinical findings in early disease and the limited progress in developing highly specific diagnostic biomarkers. Advancements in radiology, along with emerging artificial intelligence (AI)-based tools, offer new opportunities to improve the early detection of SpA [4].

Based on the primary clinical presentation, SpA can be broadly classified into two major subtypes: axial SpA (axSpA) and peripheral SpA (pSpA). The classification of axSpA includes both non-radiographic axSpA and radiographic axSpA (traditionally referred to as ankylosing spondylitis, AS), predominantly affecting the axial skeleton, particularly the sacroiliac joints (SIJ). Non-radiographic axSpA is defined by clinical features of disease in the absence of definitive sacroiliitis on radiographs and may represent an early or milder stage of radiographic axSpA, with an estimated progression rate of approximately 12% over two years [5]. Patients typically report inflammatory back pain with prolonged morning stiffness, although peripheral manifestations are also observed in 30–50% of cases [6]. The primary entities within the category of pSpA include psoriatic arthritis (PsA), reactive arthritis (ReA), and enteropathic arthritis associated with IBD, with PsA being the most common. Clinical hallmarks include arthritis, enthesitis, and/or dactylitis, though the course and treatment response differ between subtypes. For example, sustained remission is uncommon in PsA, whereas up to 60% of patients with ReA achieve remission within six months [7, 8].

Despite increasing awareness of SpA among patients and clinicians, timely diagnosis remains a major challenge [9, 10]. The insidious onset of symptoms and subtle physical findings frequently result in diagnostic delays, leading to greater functional impairment, reduced treatment efficacy, and diminished quality of life [11]. Comparable diagnostic difficulties are also observed in other systemic autoimmune diseases such as systemic lupus erythematosus, where autonomic reflex testing has been investigated as a supportive diagnostic tool to enhance clinical assessment [12]. Imaging methods—including conventional radiography (CR), computed tomography (CT), magnetic resonance imaging (MRI), and ultrasound (US)—are therefore indispensable for early detection and disease monitoring. To facilitate accurate classification and guide management, several criteria have been proposed, with the Assessment of SpondyloArthritis International Society (ASAS) criteria currently offering the best balance of sensitivity and specificity for both axSpA and pSpA [13]. Nevertheless, their application in low-prevalence populations may lead to an increased rate of false-positive diagnoses [14]. These limitations highlight the need for more advanced diagnostic tools.

Emerging research suggests that artificial intelligence (AI) can significantly enhance SpA management by improving diagnostic accuracy, supporting disease classification, predicting treatment response, and refining risk assessment. Convolutional Neural Networks (CNNs) remain the most widely applied algorithms, with U-Net models used for automated SIJ segmentation, while ResNet and DenseNet architectures have shown strong performance in detecting sacroiliitis. When combined with conventional imaging modalities, AI models have achieved diagnostic accuracy comparable to expert assessment. Although current use is limited by technical, ethical, and legal considerations, AI holds substantial promise as a complementary tool to clinical expertise, potentially enabling more personalized and efficient care [15].

This review focuses primarily on the axial form of SpA, in which imaging plays a central role in detecting inflammatory changes and monitoring structural progression. Due to the paucity of epidemiological data on pSpA, as well as the clinical heterogeneity of the diseases included in this category, the number of related clinical studies remains limited. The symptomatic overlap with other entities and the lack of specific biomarkers results in pSpA being less clearly defined than axSpA, which poses a challenge for establishing comprehensive diagnostic criteria and developing evidence-based treatment strategies for this patient population. The application of the ASAS classification criteria for pSpA, introduced in 2011, has been scarcely investigated in clinical research, with only a few exceptions [16]. The diagnostic assessment of pPsA and the role of ultrasonography (US) are briefly addressed in a separate practical section.

In this study we aimed to address recent developments in imaging of axial and peripheral form of SpA, including the critical analysis of available methods. Our study sought to discuss key considerations and challenges associated with implementing AI-based tools in clinical practice, along with potential solutions. Additionally, we intended to provide an up-to-date imaging algorithm for suspected axSpA, which can be used by clinicians.

## Search strategy

Comprehensive literature searches through PubMed, Embase, and Scopus were carried out in August 2025 following the previously published recommendations [17]. The keywords like “spondyloarthritis”, “axial spondyloarthritis”, “ankylosing spondylitis”, “psoriatic arthritis”, “radiography”, “magnetic resonance imaging”, “computed tomography”, “ultrasound”, “imaging”, “artificial intelligence”, “deep learning”, “machine learning”, “neural networks”, “radiomics” were used to obtain data that aligned with our review criteria. Each article was carefully evaluated for relevance, and its reference list was reviewed to identify additional pertinent sources. Studies published from January 1 st 2010 to August 1 st 2025 were included in the study. The analysis was limited to English documents only. The inclusion criteria comprised peer-reviewed original research articles, systematic or narrative reviews, consensus statements, and clinical guidelines related to imaging or artificial intelligence in spondyloarthritis. Exclusion criteria encompassed non-peer-reviewed materials, editorials, commentaries, conference abstracts, and case reports with insufficient imaging or AI data. Studies not involving human subjects or focusing exclusively on non-rheumatologic conditions were also excluded, as well as articles lacking methodological transparency, incomplete datasets, or unavailable full text. This narrative review followed a structured approach, and the SANRA (Scale for the Assessment of Narrative Review Articles) framework was used to guide reporting quality and ensure methodological transparency [18].

## Conventional radiography

**Fig. 1:**
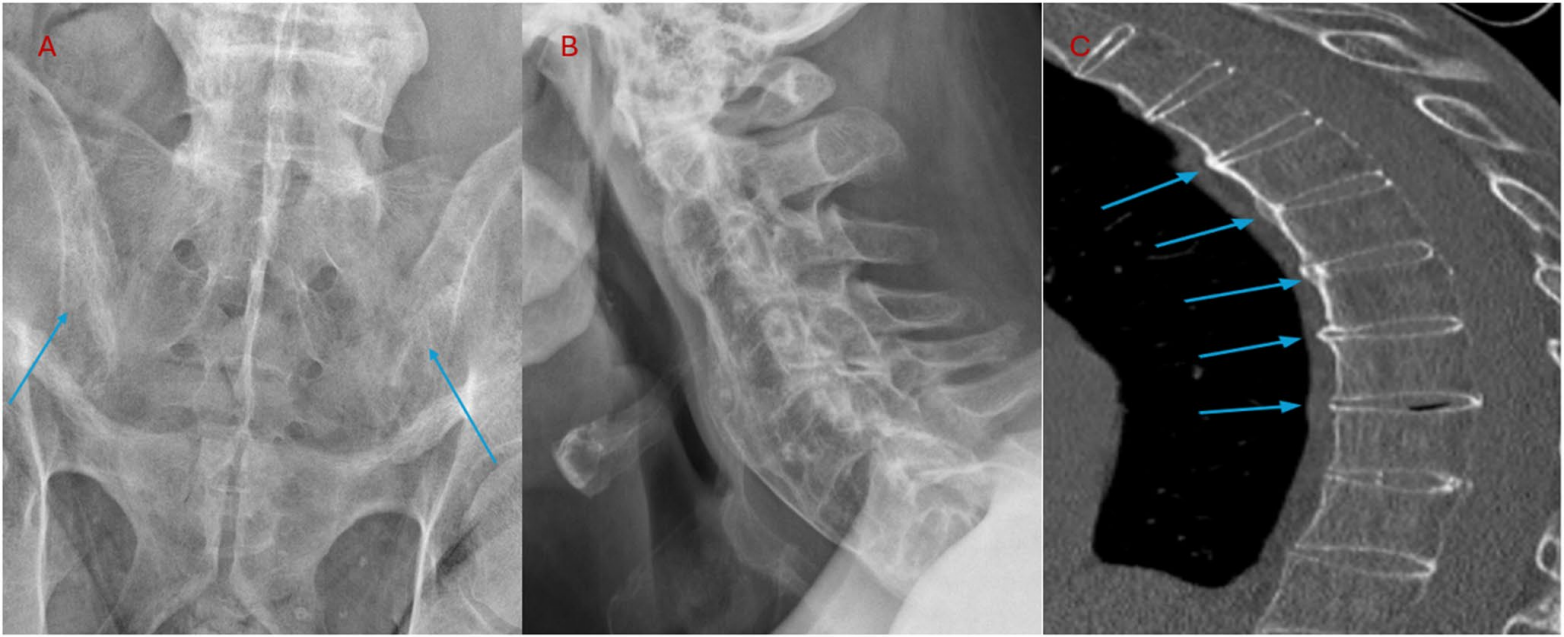
**A** Pelvic radiograph, anteroposterior view: sacroiliac joint changes consistent with ankylosing spondylitis (axial spondyloarthritis). **B** Cervical spine, lateral radiograph: complete fusion of anterior and posterior spinal elements, producing the classical “bamboo spine” appearance. **C** Thoracic spine, sagittal CT: thin vertical syndesmophytes are visible (blue arrows) in female patient with axial spondyloarthritis

Plain radiography of the SIJ typically remains the initial screening test for axSpA owing to its low cost and widespread accessibility. Radiographic damage in axSpA is most frequently assessed using standardized scoring systems such as the modified New York (mNY) criteria for the SIJ evaluation and the modified Stoke Ankylosing Spondylitis Spine Score (mSASSS) for the spine evaluation. Common radiographic findings include sclerosis, erosions, syndesmophytes and ankyloses (Fig. 1A and B). Those structural lesions may take years to develop, proving radiographs insufficiently effective in early disease. Monitoring structural progression in the spine, essential for evaluating structure-modifying treatment effects in SpA, remains challenging. Radiography assessed by the mSASSS shows limited sensitivity to change and fails to capture short-term progression (≤ 2 years) [19].

In everyday clinical practice, standard antero-posterior (AP) lumbar spine radiographs are commonly used to assess the SIJ. However, due to overlapping structures on a two-dimensional image, an accurate interpretation of pelvic radiographs could be problematic for both experienced radiologists and deep learning algorithms [20, 21]. Evidence indicates that only 30–60% of patients diagnosed with axSpA present radiographic signs of sacroiliitis described as grade 2 bilaterally or grade 3 unilaterally, according to the mNY criteria [22]. The low pooled sensitivity and specificity (66% and 67%, respectively), as well as the high rates of false positives and negatives, question the role of radiography in detecting active inflammation [23]. Moreover, the number of patients with suspected axSpA benefiting from CR appears to be limited, as the bone marrow edema (BME), an important marker of early lesions in axSpA, remains undetected in this type of imaging [24].

There is an ongoing debate about whether CR should be replaced by cross-sectional imaging or more advanced diagnostic techniques which achieve better sensitivity, specificity, and reliability. In centers with access to MRI, particularly in young patients with short symptom duration, plain radiographs may add little diagnostic value. Nevertheless, radiography remains useful for documenting advanced structural changes and in settings with limited MRI availability [23].

Several studies have investigated the value of digital tomosynthesis (DTS) in detecting structural damage in SpA. This technique is well established and widely recognized as a breast cancer screening tool, particularly in women with dense breast tissue, where lesion visibility on standard mammography is often limited [25]. DTS involves acquiring two-dimensional radiographic images during a single sweep of the X-ray tube, which are then reconstructed into sectional pseudo-tomographic slices. By limiting anatomical overlap, DTS provides better visualization of complex structures such as the SIJ [26]. In musculoskeletal imaging, DTS has gained significance in detecting early structural lesions of the SIJ in patients with non-radiographic axSpA [27]. Recent study demonstrates the superiority of DTS over CR in detecting sacroiliitis, achieving better sensitivity, specificity and accuracy (87%, 91%, 77%, respectively, for DTS and 60%, 84%, 44% for radiography) [28]. Moreover, DTS has proven useful for assessing spinal lesions in patients with AS, as it can depict subtle vertebral and facet joint damage that is often overlooked by radiographs. Evidence also demonstrates that DTS allows for a more accurate evaluation of AS-related spinal damage using the mSASSS scoring system [29]. However, distinguishing structural lesions caused by inflammatory versus degenerative processes may be more challenging with DTS than with CT. Due to its lower sensitivity, DTS is inferior to CT in detecting structural damage of the SIJ. Notably, the mean effective dose for DTS is significantly lower than that of CT, which suggest that it could serve as an alternative to CR in evaluating structural changes in axSpA [28].

Aiming to enhance the diagnostic efficiency of CR, several AI-based algorithms have been developed. Bressem et al. [30] used ResNet-50 architecture for detecting sacroiliitis on conventional radiographs, achieving excellent result with an area under the curve (AUC) of 0.97 for the validation and 0.94 for the test. Heterogenous training dataset used in their research resulted in good generalizability of the protocol, which was demonstrated on test data, reaching an expert-level judgement. However, the accuracy of this model in diagnosing non-specific back pain suspected of SpA was not established, since all patients included in the study were diagnosed with axSpA. Another successful accomplishment was the development of a deep learning algorithm predicting active sacroiliitis based on SIJ radiographs conducted by Turk et al. [31]. JointNet model used in the study identified active inflammation with the accuracy of 90.2%, outperforming human observers (accuracy of 54–64%). Dorfner et al. [32] investigated the impact of incorporating anatomical awareness into deep learning models to enhance generalizability and predict disease progression. The anatomy aware model outperformed the standard model, achieving higher accuracy in detecting radiographic sacroiliitis. In addition, it demonstrated the ability to identify patients at risk of disease progression. However, it remains unclear whether the model genuinely predicted the development of radiographic sacroiliitis or if it merely detected subtle early-stage features that human observers might consider inconclusive. In addition, Meng et al. [33] developed and validated a deep learning model for the automated grading of radiographic sacroiliitis. With the assistance of the algorithm, the image grading accuracy of the two radiologists showed significant improvement, from 75% and 74% to 89% and 81%, respectively. Nevertheless, authors emphasize the necessity of thorough external validation to confirm the applicability of these methods in clinical practice.

## Computed tomography

According to European League Against Rheumatism (EULAR) guidance, in the absence of detectable abnormalities on CR and in situations where MRI cannot be conducted or is contraindicated, CT may provide complementary information on structural damage, as it is considered the gold standard for the visualization of such lesions [34]. It provides high specificity (reaching up to 93.3%) [23] in the assessment of cortical and trabecular bone changes which may go unnoticed on MRI [35]. However, BME of the SIJ, the principal indicator of active inflammation, is not detectable with conventional CT. Given its high costs compared to CR and significant ionizing radiation exposure, conventional CT is not frequently selected to assess structural changes in SpA [34]. Concerns about radiation exposure focus on the cumulative dose from repeated imaging. Current evidence indicates that oblique coronal CT reduces gonadal radiation compared to axial CT and should be considered when MRI is unavailable. However, the effective radiation dose remains substantial, limiting its use in patients undergoing long-term monitoring [36, 37]. Recent technological advancements such as low-dose CT (ldCT), ultra-low-dose CT (uldCT) and dual-energy CT (DECT) have expanded the potential applications of this imaging technique for clinical use and scientific research. From a clinical perspective, minimizing radiation exposure is particularly important in younger patients and those requiring serial follow-up, as even modest cumulative doses may increase the long-term risk of malignancy and gonadal damage. Consequently, ldCT and uldCT protocols are increasingly preferred over conventional CT, offering a more favorable balance between image quality and patient safety.

Low-dose CT and uldCT have emerged as useful methods outperforming CR for lesion detection, enabling radiation dose reduction while preserving sufficient image quality [28, 29] The effective radiation dose for ldCT of the spine and the SIJ is usually < 1 mSv, comparable to CR. Advances in AI-based image reconstruction methods allow further dose reduction, as the uldCT is associated with only 10% of the radiation dose of a conventional CT. In recent years, ldCT has gained importance, particularly for assessing structural progression. The CT Syndesmophyte Score (CTSS) enables quantitative evaluation of syndesmophytes, and studies demonstrate that ldCT is more sensitive than radiography in detecting new bone formation, especially in the thoracic spine (Fig. 1C).

DECT, also known as spectral CT, enables the visualization and quantification of the extent of BME by measuring relative water and calcium concentration in bone. Evidence indicates that DECT is comparable to MRI for identifying BME, yet MRI continues to demonstrate the highest sensitivity for detecting synovitis, enthesitis and capsulitis [39]. However, contrary to the aim of reducing radiation exposure, DECT protocols employ radiation doses equivalent to, or even greater than, those associated with conventional CT. For this reason, as well as due to the occurrence of artifacts that complicate image interpretation, the utility of this method remains confined to research applications [38]. Table 1provides an overview of the key characteristics, applications, and limitations of ldCT and the CTSS in the assessment of axial spondyloarthritis.

**Table 1:** Low-dose CT (ldCT) and CT syndesmophyte score (CTSS) in axial spondyloarthritis

Aspect	Details	Notes
Radiation dose	Effective dose usually < 1 mSv	Comparable to CR, lower than standard CT
Coverage	Thoracic spine	Thoracic spine particularly important for new bone formation
Scoring system	CTSS: semi-quantitative, vertebral quadrants assessed for syndesmophytes	Higher sensitivity vs. radiography
Applications	Detection and monitoring of syndesmophyte progression	Useful in clinical trials and long-term follow-up
Limitations	Radiation exposure, limited availability	Not recommended for routine diagnosis in early disease

*CR* conventional radiography, *CT* computed tomography, *CTTS CT* syndesmophyte score

Several clinical studies have explored the application of deep learning to CT for diagnosing SpA. Zhang et al. [40] developed a fully automated method for segmenting the SIJ and grading sacroiliitis associated with AS on CT, aiming to reduce inter-observer variability that often complicates assessment. The 3D CNN model, trained to grade sacroiliitis according to the mNY criteria, achieved high performance, particularly for class 0 (equivalent to grades 0-I of the mNY criteria, indicating the absence of definite sacroiliitis) and class 2 (equivalent to grades III-IV of the mNY criteria, representing moderate-to-severe sacroiliitis). Its performance was lower for class 1 (equivalent to grade II of the mNY criteria, indicating mild sacroiliitis), yet it still outperformed the senior radiologist. The study had certain limitations, as the definition of ground truth depends on a radiologist consensus, which may introduce bias. The authors emphasize the need to further optimize the model through slice-based grading and by expanding the sample size to enhance its potential for clinical application.

Aiming to increase diagnostic performance in CT, Van den Berghe et al. [41] developed and validated a multicenter deep learning model to identify structural lesions associated with sacroiliitis. The algorithm demonstrated strong diagnostic accuracy for detecting erosions and ankyloses, both on slice-by-slice and patient level. However, it has not yet been tested prospectively within clinical workflow settings or in patients without a suspicion of sacroiliitis.

Shenkman et al. [42] introduced an automated algorithm for the diagnosis and grading of sacroiliitis on CT scans as incidental findings, in patients undergoing the evaluation of lower back pain. The algorithm demonstrated strong performance, achieving accuracy of 92% and an AUC of 0.97 for binary detection of sacroiliitis, although it was not further validated on external datasets.

## Magnetic resonance imaging

MRI remains the reference standard for detecting early inflammatory and structural changes in axial spondyloarthritis, allowing simultaneous assessment of BME, erosions, fat metaplasia, and ankylosis [20]. In 2024, ASAS together with the SpondyloArthritis Research and Treatment Network (SPARTAN) proposed a consensus minimal protocol for MRI of the SIJ, designed to standardize image acquisition and improve comparability between centers [43]. An international consensus recommends a minimum four-sequence MRI protocol for accurate evaluation of inflammation, structural lesions, and the bone-cartilage interface. The reference standard includes three semicoronal sequences (aligned with the posterior surface of the S2 vertebral body) and one semiaxial sequence (perpendicular to semicoronal). Each of the semicoronal sequences is serving a distinct imaging purpose: T1-weighted spin echo for identifying fat signal alternations associated with structural damage, short tau inversion recovery (STIR) or T2-weighted with fat suppression (T2FS) sensitive for detecting active inflammation, and high resolution or thin slices T1-weighted sequence for the precise visualization of the bone-cartilage interface and detection of bone erosions. The semiaxial T2FS sequence is designed to optimally visualize BME, providing a complementary view of inflammatory changes. Importantly, contrast-enhanced sequences are not usually necessary and should be used only for unusual circumstances. According to expert agreement, this protocol should not be used for screening purposes, as it has been designed for the diagnostic evaluation of axSpA-related sacroiliitis and its differential diagnoses [44].

### Updated definitions of spinal lesions on MRI

**Fig. 2:**
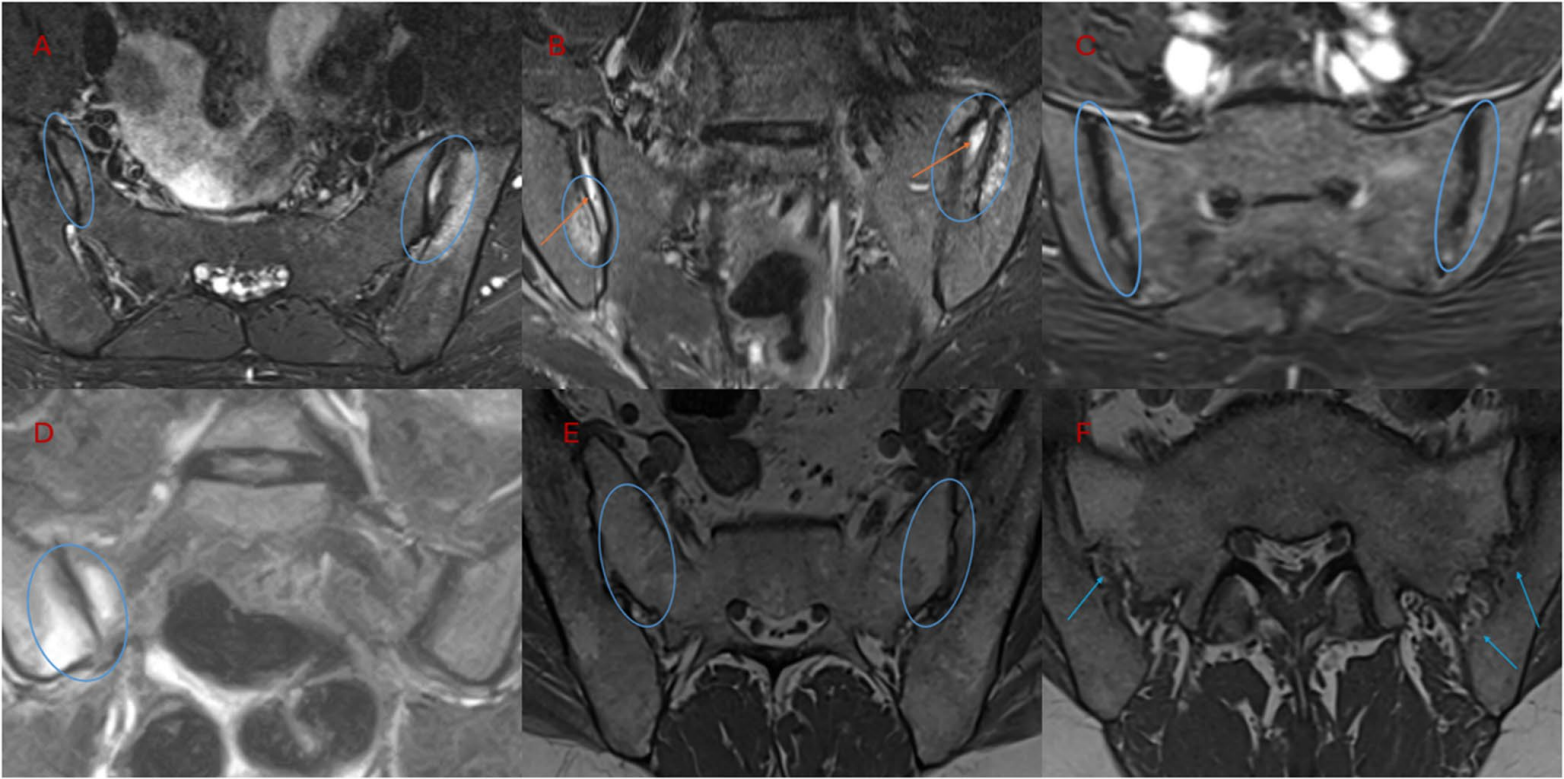
**A** Axial STIR sequence: sacroiliitis in axial spondyloarthritis showing bilateral high signal intensity consistent with bone marrow edema, more pronounced on the left side. **B** Axial STIR sequence: postpartum bone marrow edema at the sacroiliac joints (loop), with joint distraction (arrows) and fluid in the joint space. **C** Axial STIR sequence: osteitis condensans ilii with bilateral iliac sclerosis and mild sacroiliac joint irregularity, without associated bone marrow edema. **D** Axial STIR sequence: sacroiliac joints in a young female with inflammatory back pain. MRI confirms classical right-sided active sacroiliitis with subchondral bone marrow edema, consistent with early axial spondyloarthritis. In advanced disease, changes typically become bilateral and symmetrical. **E** Axial T1 sequence: fat metaplasia presenting as homogeneously bright signal in the subchondral bone. **F** Axial T1 sequence: classical bilateral iliac erosions (arrows), characterized by loss of cortical bone definition

In 2022, the ASAS MRI working group updated and validated definitions of inflammatory and structural lesions in the spine. Inflammatory changes include BME easily seen on at least two consecutive slices of an MRI scan, while structural changes include erosions, fat metaplasia, and syndesmophytes documented on T1 sequences. The update specifies more precisely where lesions are located (central vs. lateral slices), how far they extend (vertebral corner vs. vertebral endplate) and how extensive they must be (minimum number of sagittal slices on which they must be visible). In addition, new terms were introduced to describe lesion “maturity”, distinguishing monomorphic from dimorphic vertebral corner lesions. Incorporating these definitions into routine reports facilitates standardization and comparability across studies. Figure 2A–F shows characteristic MRI features of axSpA and its differential diagnoses.

### Inflammatory lesions

Inflammatory lesions on MRI are characterized primarily by BME, which appears hyperintense on STIR or T2 fat-suppressed sequences and hypointense on T1-weighted images. According to ASAS definitions, BME should be visible on at least two consecutive sagittal slices for vertebral body lesions, whereas lateral lesions, facet joint lesions and posterior element lesions may be recorded if present on a single slice, provided that their morphology and distribution are typical for axSpA. Vertebral corner inflammation may appear as either a monomorphic lesion, where the increased signal reaches the cortical margin, or a dimorphic lesion, where the signal spares the corner cortex but extends to both the endplate and the anterior or posterior vertebral border, sometimes accompanied by structural changes such as fat, sclerosis or erosion.

### Structural lesions

Structural lesions on MRI include erosions, fat metaplasia, syndesmophytes or ankylosis. Erosions appear as sharply demarcated cortical defects with low signal intensity on T1-weighted images and corresponding loss of cortical integrity. Fat metaplasia presents as homogeneously hyperintense areas on T1 sequences, typically located in the vertebral corners and representing fatty replacement of previous inflammatory lesions. Syndesmophytes and ankylosis manifest as bony bridges across intervertebral disc spaces or facet joints, best visualized on T1-weighted sequences. Sclerosis may also be observed as low signal on all sequences, but it is considered insufficiently specific to be included among axSpA-defining structural lesions and should be interpreted with caution in the context of possible degenerative disease.

### Reporting recommendations

A structured MRI report should describe the presence, number, and distribution of BME lesions, identify structural abnormalities such as erosions, fat metaplasia, and syndesmophytes or ankylosis, and assess both anterior and posterior vertebral elements. It should also include an interpretation of potential alternative explanations for the observed findings, such as degenerative changes or post-traumatic alterations. The key elements of a standardized MRI reporting framework, based on the ASAS MRI Working Group 2022 recommendations, are summarized in Table 2.

**Table 2:** Structured MRI spine report in axial spondyloarthritis (based on ASAS MRI WG 2022)

Category	Items to report
Inflammatory lesions	Presence/absence of BME visible on ≥ 2 consecutive sagittal slices (except lateral, facet joint and posterior element lesions, which may be recorded on a single slice) Distribution (cervical/thoracic/lumbar) Posterior element involvement (costovertebral, facet, spinous processes)
Structural lesions	Erosions (location, number, distribution) Fat metaplasia (corner/non-corner, extent) Syndesmophytes/ankylosis (level, bridging across disc space)
Additional considerations	Co-existing degenerative lesions (Modic, Schmorl’s nodes, osteophytes) Other differential diagnoses (trauma, infection, metabolic bone disease) Overall impression: consistent/not consistent with axSpA Sclerosis (low signal T1/STIR, low specificity, often degenerative)

*BME* bone marrow edema, *STIR* short tau inversion recovery, *axSpA* axial spondyloarthritis

### Differential diagnosis of sacroiliac joint bone marrow edema

Interpretation of sacroiliac joint MRI requires careful differentiation between BME caused by axial spondyloarthritis and a variety of non-specific mimics. Postpartum changes, osteitis condensans ilii, sports-related stress reactions, infectious sacroiliitis, and calcium pyrophosphate deposition disease may all produce signal alterations resembling inflammatory lesions but differ in symmetry, location, and associated structural features. Recognizing these patterns is essential to prevent misinterpretation and ensure diagnostic accuracy. Characteristic imaging features of these conditions are summarized in Table 3.

**Table 3:** Differential diagnosis of sacroiliac joint bone marrow edema

Category	Typical features	Notes
Postpartum edema	Symmetric, sacral-sided BME, resolves within 6–12 months	Important to distinguish from early axSpA in young women
OCI	Well-demarcated iliac-sided sclerosis, triangular shape, absence of erosions	Frequently seen in multiparous women, non-inflammatory
Sports-related stress changes	Focal BME at anterior joint margins, often unilateral or asymmetric	Related to mechanical overload in athletes or physically active individuals
Infectious sacroiliitis	Unilateral/asymmetric BME, erosions, possible joint effusion or abscess	Requires urgent differentiation, clinical and laboratory correlation essential
CPPD	Calcifications and cartilage deposits, sometimes associated with periarticular BME	Often co-exists with degenerative changes, older patients

*BME* bone marrow edema, *axSpA* axial spondyloarthritis, *OCI* osteitis condensans ilii, *CPPD* calcium pyrophosphate deposition disease.

### Artificial intelligence in MRI

Challenges in detecting inflammatory changes and evaluating their compatibility with axSpA features have underscored the need of employing enhanced MRI-based techniques [43]. AI-driven approaches have previously been applied to various MRI datasets, including rheumatological imaging applications. According to recent studies, deep learning models are achieving progressively greater results in detecting sacroiliitis, attaining accuracy comparable to, or even exceeding, that of radiologists. Notably, recently developed algorithms have shown effectiveness in leveraging clinical factors (e.g., back pain, HLA-B27 status and extra-articular symptoms) to detect axSpA [44].

Latest studies have demonstrated significant advancements in applying deep learning techniques to MRI for diagnosing axSpA. Zhang et al. [46] developed a CNN-based model integrating multi-sequence MRI and clinical data, attaining accuracy of 85.6% and AUC of 0.91 in diagnosing sacroiliitis. Aiming to improve the diagnostic performance of MRI, Chan et al. [47] introduced a deep learning model for detecting active spinal inflammation on STIR MRI sequence, using ‘fake-color’ images to mimic adjacent MRI slices. The performance of the developed U-Net model was similar to that of a general radiologist, demonstrating comparable sensitivity and specificity.

Xie et al. conducted a multicenter study, introducing a deep learning model based on ResNet50 which exceeded the ASAS criteria with better sensitivity (87.8% vs. 42.4%), accuracy (78.7% vs. 56.9%) and AUC (0.858 vs. 0.650) [48]. Nevertheless, these promising findings require careful consideration, as there are still not enough prospective multicenter trials to support their validity and applicability [15].

Several studies have investigated the potential of MRI-based synthetic CT (sCT) for visualizing bony structures. This novel technique generates CT-like images derived from an axial 3D T1-weighted radiofrequency spoiled multiple gradient-echo (T1MGE) MRI sequence using a deep learning-based image synthesis. In the assessment of axSpA, sCT is emerging as a radiation-free alternative to conventional CT for evaluating structural lesions. The study by Jans et al. [49] demonstrated that sCT achieved higher accuracy than T1-weighted MRI in detecting erosions (94% vs. 86%), sclerosis (97% vs. 81%), and ankylosis (92% vs. 84%). Further research on the diagnostic utility of sCT was conducted by Willesen et al. [50], who evaluated its performance in detecting spinal new bone formation in patients with axSpA. Using ldCT as the reference standard, sCT achieved specificity comparable to that of ldCT, and much higher sensitivity than radiography (67% vs. 13%). These results suggest that sCT has the potential to become valuable tool for detecting and monitoring structural damage in axSpA.

## Ultrasound

Peripheral manifestations in SpA are commonly assessed using ultrasound (US), which allows real-time evaluation of inflammatory changes such as enthesitis, synovitis and capsulitis without radiation exposure [51, 52]. To date, its contribution has been secondary, given that the diagnosis of pSpA relies predominantly on clinical assessment, a comprehensive personal and family record, prior infections, and SpA-related comorbidities [53]. However, recent advances have highlighted the importance of combining imaging findings with clinical evaluation to improve diagnostic accuracy. Di Matteo et al. [54] successfully developed the DEUS (Defining Enthesitis on Ultrasound in Spondyloarthritis) Enthesitis Score (DEI), which combines ultrasound findings with clinical examination for the evaluation of enthesitis in patients with SpA. The DEI models show a stronger correlation with C-reactive protein levels and structural damage at the entheses than clinical examination alone. This integrated approach holds great potential for more comprehensive assessment of enthesitis in SpA patients, combining objective imaging data with clinical observations and patient-reported outcomes.

Although both EULAR recommendations and ASAS criteria do not include US as a reliable tool for diagnosing axSpA-related sacroiliitis, recent studies suggest that it may be used in the diagnostic evaluation of AS. Zhu et al. [55] demonstrated that US, especially color Doppler US, represents a robust and reproducible method for diagnosing AS with an AUC of 0.93. However, analysis revealed that the accuracy of AS activity assessment using this modality is not ideal (AUC of 0.64). Despite the promising results, US has several limitations such as low specificity (54%), lack of universal scoring system and lower inter-observer reliability compared to modalities like MRI or CT, making it difficult to standardize and effectively train AI algorithms.

Research conducted by Castro-Corredor et al. [56] indicates that machine learning models can quantify enthesitis using the Madrid Sonographic Enthesitis Index (MASEI) score and detect subclinical inflammation. By standardizing enthesis evaluation and minimizing interobserver variability, these algorithms may improve diagnostic precision and facilitate disease monitoring in both PsA and ReA. Furthermore, the combination of Doppler US with AI-assisted vascular signal analysis may enable more reliable assessment of inflammatory activity in peripheral joints and tendons. Interestingly, the machine learning- assisted quantification of synovial fluid and hypertrophy in ultrasound, demonstrated in rheumatoid arthritis by Hemalatha et al. [57], could potentially be applied in SpA to reduce inter- and intra-observer variability and improve diagnostic accuracy. Although current data remain limited, the integration of ultrasound with AI-driven image analysis represents a promising direction for the assessment of pSpA. Future research should focus on developing standardized scoring systems, large annotated datasets, and multimodal frameworks that combine ultrasound findings with MRI or clinical parameters.

## Up-to-date imaging algorithm for suspected axial spondyloarthritis

The choice of a first-line imaging modality in suspected axSpA is often influenced by accessibility and cost-effectiveness. However, recognizing the distinct clinical features of patients with radiographic axSpA versus non-radiographic axSpA may further guide the selection of the most appropriate imaging modality for each patient. Evidence indicates that MRI should be considered the first-line technique in younger patients presenting with early-stage symptoms. Conversely, CR is recommended as the initial diagnostic method in older patients with longer history of back pain, with thresholds for age and symptom duration established at 33.5 and 4.1 years, respectively [58].

It is well acknowledged that CT provides the highest accuracy in visualizing structural lesions, which are often crucial for differential diagnosis [23]. Nevertheless, sacroiliitis detected on CT has not been included in the ASAS classification criteria, likely due to concerns regarding the associated high radiation exposure. Advances in imaging technology have enabled SIJ CT to be performed with a reduced radiation dose, and consequently, low-dose CT has emerged as a reliable modality with high specificity for axSpA. Recent findings suggest that ldCT may be useful even in early disease, particularly when CR is inconclusive and MRI is contraindicated or unavailable [59].

An important marker of early lesions in axSpA, BME of the SIJ, is typically assessed using MRI. Despite its high sensitivity and specificity in detecting early inflammatory changes, limited accessibility and contraindications have led to the investigation of DECT as a potential alternative. However, recent data suggest that this technique is unsuitable for routine clinical practice. Obtaining diagnostically reliable images is associated with high radiation doses, which should be avoided, particularly in young patients requiring serial follow-up evaluations. Consequently, MRI remains the reference standard for visualizing inflammatory bone marrow lesions [60].

The contemporary imaging algorithm for suspected axSpA reflects a balance between accessibility, diagnostic accuracy, and patient safety (Table 4).

**Table 4:** Comparison of imaging modalities in the diagnosis and monitoring of axial spondyloarthritis

Modality	Sensitivity (pooled/range)	Specificity (pooled/range)	Radiation (mSv)	Relative cost	Utility (baseline/monitoring)
CR	55 (12–83%)	87 (62–100%)	0.15 ± 0.10	$	Baseline: structural damage; Monitoring: limited
MRI	35–91%	75–90%	–	$ $ $	Baseline: active inflammation + structural damage; Monitoring: first-line modality
ldCT	77%	96%	0.42 ± 0.18	$ $	Baseline: structural damage Monitoring: limited
DECT	79% (55–91%)	92% (89–93%)	2–5	$ $	Baseline: BME; Monitoring: limited
US	86% (58–100%)	54 (35–100%)	–	$	Baseline and Monitoring: primary modality in pSpA, adjunctive role in axSpA

*CR* conventional radiography, *MRI* magnetic resonance imaging, *ldCT* low-dose computed tomography, *DECT* dual-energy computed tomography, *BME* bone marrow edema, *US* ultrasonography, *pSpA* peripheral spondyloarthritis, *axSpA* axial spondyloarthritis

**Fig. 3:**
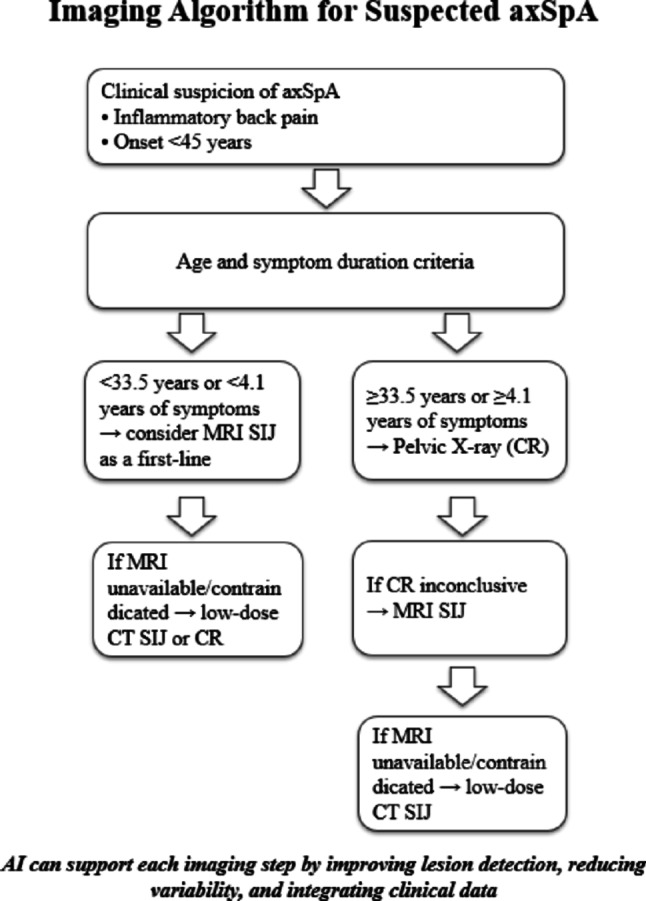
Imaging algorithm for the diagnostic workup of suspected axial spondyloarthritis

While conventional radiography remains the most widely available initial test, particularly in healthcare systems where MRI access is limited and reimbursement pathways are restrictive, its low sensitivity in early disease often delays diagnosis. MRI, with superior ability to detect active inflammation, is increasingly recommended as a first-line modality in younger patients with shorter symptom duration, whereas low-dose CT provides valuable complementary assessment of structural damage when MRI is inconclusive or contraindicated. Nevertheless, the variability in imaging availability, radiation exposure, and the lack of standardized scoring systems for ldCT and DECT continue to hinder their routine use. Recent advances in AI offer promising opportunities to enhance diagnostic accuracy, reduce interobserver variability, and integrate imaging with clinical and laboratory data for more individualized evaluation. However, these technologies require external validation and infrastructural adaptation before widespread clinical implementation. Overall, a multimodal, patient-centered, and AI-assisted imaging strategy appears to be the most effective direction for improving early recognition and management of axSpA. A summary of the proposed diagnostic imaging workflow is presented in Fig. 3.

## Peripheral spondyloarthritis

Although less frequently addressed than the axial form, pSpA represents a clinically important subgroup of SpA with distinct diagnostic challenges [61]. The classification of pSpA is primarily based on the ASAS criteria, which incorporate peripheral arthritis, enthesitis, and dactylitis as entry points, followed by additional clinical, laboratory, and imaging features. HLA-B27 positivity, a family history of SpA, and the presence of psoriasis or IBD may strengthen diagnostic confidence. Extra-articular manifestations such as uveitis or skin and bowel disease often aid in clinical recognition. Unlike axSpA, imaging plays a supporting rather than central role in pSpA diagnosis, but it is increasingly valuable for early detection and disease monitoring.

Ultrasound has emerged as the most practical imaging tool in pSpA, offering real-time, non-invasive visualization of synovitis, tenosynovitis, bursitis, and enthesitis. Power Doppler US improves sensitivity in detecting active inflammation by identifying hypervascularization at entheses or within synovium. Several scoring systems have been proposed, with the MASEI being the most widely investigated [62]. US is also particularly helpful in differentiating inflammatory enthesitis from mechanical or degenerative changes, which can be challenging in clinical examination alone.

MRI may be applied in selected cases, especially for atypical presentations or when deeper entheses (e.g., pelvic or hip entheses) are affected and are inaccessible to US. MRI provides superior sensitivity in detecting early inflammatory changes, including BME, and can be used to assess the extent of soft tissue and joint involvement [63].

Recent advances in AI have also begun to influence diagnostic approaches in pSpA [64]. Early studies suggest that deep learning algorithms can automatically quantify sonographic enthesitis scores or detect subtle inflammatory changes on MRI, potentially reducing observer variability and improving diagnostic consistency. However, standardized protocols and external validation in multicenter cohorts are still lacking.

Taken together, the diagnosis of pSpA remains primarily clinical, supported by targeted imaging, laboratory testing, and the exclusion of differential diagnoses such as crystal arthropathies or mechanical enthesopathies. As imaging and AI-based methods continue to evolve, they may help overcome current limitations in sensitivity, specificity, and interobserver reliability, ultimately facilitating earlier recognition and more personalized management of pSpA.

## Challenges and future directions

Despite significant progress which has been achieved over the past decades, diagnosing SpA remains challenging due to its insidious course and the absence of highly specific diagnostics biomarkers, often resulting in delayed diagnosis and worse clinical outcomes [4]. The well-established imaging modalities face certain limitations which may restrict their application in specific circumstances. Radiography, while widely accessible and cost-effective, lacks accuracy in early disease. MRI remains the gold standard in evaluating acute inflammatory changes, yet its use may be limited by availability or patient contraindications. Emerging diagnostic techniques such as ldCT have demonstrated the potential to improve medical imaging analysis and provide multimodal diagnostic strategy. However, the absence of a wide standardized scoring protocol for ldCT, along with concerns about radiation exposure associated with DECT, poses challenges for their use in routine clinical setting [43].

Recently, deep learning algorithms have become powerful instruments increasing diagnostic accuracy, as well as integrating imaging and clinical data for prognostic modeling. The integration of imaging findings with clinical and electrophysiological data has already proven useful in cardiology, as shown by the development of composite scores such as the CAR₂E₂ Score for left ventricular hypertrophy detection, which combined chest radiograph, electrocardiography, and clinical parameters [65]. A similar multimodal strategy may enhance diagnostic accuracy in SpA when supported by AI algorithms.

While recent studies provide valuable insights into the role of imaging and AI in SpA, most available evidence derives from single-center or retrospective designs, limiting the generalizability of their conclusions. Methodological heterogeneity across imaging protocols, AI architectures, and reference standards further complicates comparison between studies and may account for the wide variation in reported diagnostic accuracy. Few investigations have evaluated clinical outcomes or cost-effectiveness, which are crucial for translating these advances into routine practice. Therefore, future research should not only focus on technical performance but also on clinical validation, external reproducibility, and real-world impact on diagnostic decision-making. Collectively, these factors underscore the need for harmonized imaging standards and the integration of advanced computational tools to improve diagnostic reliability and facilitate translation into clinical care.

The successful implementation of AI-based techniques in clinical environments encounters several obstacles. Imaging protocols, patient populations and study designs differ greatly across research centers, presenting a challenge in establishing their effectiveness. Significant heterogeneity in data formats, such as variations in image resolution parameters or post-processing techniques can limit the algorithm’s ability to accurately analyze information and hinder the deployment of AI models. The absence of robust external validation leads to poor generalizability, making it one of the major challenges in evaluating and implementing AI models [66]. This underscores the need for prospective multicenter trials to ensure validity of the obtained results. External test sets, calibration, and generalization of models are crucial, along with awareness of systematic biases. This involves testing the algorithms on sufficiently large datasets sourced from institutions other than those involved in model development. However, the many of software used in clinical medicine is proprietary and not freely accessible, which restricts its use and limits opportunities for collaboration. Conversely, open-source platforms promote cooperative development and accelerate the progress of AI technologies, offering a promising pathway to wider adoption of AI in healthcare. Open-access imaging databases align with principles of cost-effectiveness and facilitate collaboration between clinicians and researchers [67].

The use of deep learning in diagnostic evaluation also raises important concerns such as accountability for AI faults and risk of patient data breaches [68]. Liability for medical errors associated with the use of AI in clinical practice is still unclear, and legal frameworks are evolving. Although medical malpractice regulations vary between countries, there is general agreement that any AI tool should be used in accordance with the recognized standard of care [69]. The integration of AI into clinical practice must evolve within a structured environment. The European Union (EU) Artificial Intelligence Act (AI Act) proposed by the European Commission is designed to guide the transparent and responsible adoption of AI in the EU [70]. AI technologies in healthcare commonly require access to sensitive patient health information, exposing them to potential cyber threats. Cybersecurity risks necessitate careful evaluation of new or untested software, which can impede the deployment of AI solutions in clinical workflows. Effectively responding to these challenges also involves substantial investment in secure systems and staff training [71]. To address these ethical, economic, and legal challenges, a combined framework integrating open-source model validation, harmonized data-sharing policies, transparent regulatory oversight, and sustainable funding mechanisms is needed. Such coordinated efforts would facilitate the responsible and equitable implementation of AI technologies in rheumatologic imaging.

Costs of deployment, incorporation with Picture Archiving and Communication System (PACS), and medico-legal issues remain major challenges. The successful integration of AI into clinical practice can be achieved through the deployment of an open-source PACS-AI platform. This strategy is designed to enable the incorporation of AI models with available diagnostic imaging datasets. By adhering to Digital Imaging and Communications in Medicine (DICOM) standards, the PACS-AI platform facilitates interoperability between systems used to store, process, and send medical images. The aim is to develop a broadly available strategy for the deployment of AI models in clinical practice, which also ensures adherence to regional security and data protection standards.

The successful adoption of AI depends on its economic feasibility. A comprehensive cost assessment should include trial availability, setup expenses and ongoing technical assistance [67]. However, recent data suggest that health economic evaluations do not keep up with the rapid technological progress, which may hinder the adoption of AI tools in clinical practice [72]. Moreover, establishing an optimal balance between sensitivity and specificity is essential to ensure both clinical utility and long-term cost-effectiveness of AI-based diagnostic models. Improving AI’s sensitivity may increase healthcare expenses and unnecessary referrals, as higher sensitivity is often accompanied by a decline in specificity, leading to more false-positive results and additional diagnostic testing. Conversely, excessively stringent thresholds that favor specificity may reduce sensitivity and contribute to missed early cases [73]. Recent multicenter studies suggest that models combining SIJ MRI with clinical data outperform image-only models, but external validation in clinical practice is still pending.

In radiographic imaging, AI-based algorithms such as ResNet and DenseNet have achieved expert-level accuracy in detecting and grading sacroiliitis according to the mNY criteria. These models can identify both active and structural lesions, improving interobserver reliability and diagnostic reproducibility. Importantly, deep learning systems incorporating anatomical awareness have demonstrated potential for predicting radiographic progression, suggesting that AI could assist not only in diagnosis but also in prognostication. In computed tomography, fully automated CNN models have been applied for 3D segmentation of the sacroiliac joints and quantitative grading of erosions and ankylosis. Low-dose and ultra-low-dose CT combined with AI-driven reconstruction further reduce radiation exposure while maintaining image quality, creating an opportunity for safer and more precise assessment of structural lesions.

AI has found particularly valuable applications in MRI, the current gold standard for early detection of inflammatory changes in axial SpA. Deep learning algorithms based on U-Net and ResNet architectures can automatically identify BME, fat metaplasia, and erosions, often reaching sensitivity and specificity comparable to expert radiologists. Multisequence MRI models integrating T1-weighted, STIR, and T2 fat-suppressed images have been developed to classify disease activity and structural damage. Recent approaches combining MRI data with clinical variables (e.g., HLA-B27 status, inflammatory back pain, or extra-articular features) outperform image-only models, reflecting the value of multimodal analysis. Additionally, synthetic CT generated from MRI sequences using deep learning allows visualization of cortical bone with accuracy similar to low-dose CT, providing a radiation-free alternative for structural assessment. The key considerations for the application of AI in imaging of axSpA, summarizing the main domains, their associated challenges, and the implications for clinical practice are presented in Table 5.

**Table 5:** Key considerations for artificial intelligence in imaging of axial spondyloarthritis

Domain	Key points	Implications
Validation	External datasets required for reliable performance	Avoids overfitting to single-center data
Calibration	Models must be calibrated to predict true probabilities	Improves clinical interpretability
Generalization	Performance varies across scanners, protocols, populations	Requires multicenter testing
Bias	Potential for algorithmic bias (age, sex, ethnicity)	Critical for equitable implementation
Integration	Deployment in PACS, workflow integration, regulatory approval	Major barrier for clinical adoption
Cost-effectiveness	Implementation and maintenance costs need evaluation	Essential for health system adoption

*PACS* picture archiving and communication system

### Limitations

This review is narrative in nature and, despite a comprehensive literature search, may be subject to publication and selection bias. The heterogeneity of study designs and lack of standardized outcome measures across included studies limit the ability to perform quantitative synthesis. Nevertheless, this structured review highlights key advances and identifies gaps requiring further research.

## Conclusions

Recent advances in imaging and AI technologies hold promise for more effective diagnostic strategies in SpA. Multisequence MRI, ldCT as an alternative to conventional CT, and AI-driven image analysis emerge as the leading approaches for enabling more precise detection of inflammation and structural lesions. The use of these technologies should follow standardized protocols, to ensure consistency and maintain diagnostic accuracy. Collectively, these innovations may contribute to earlier recognition, precise monitoring, and personalized therapeutic approaches. For clinical practice, MRI may be considered as the preferred modality for early detection of sacroiliitis in younger patients with a short symptom duration, while conventional radiography remains the recommended first-line test in many diagnostic algorithms. Low-dose CT may be used in selected situations, particularly when MRI results are inconclusive, when MRI is contraindicated, or when detailed structural assessment is required. AI-assisted image interpretation may, in the future, be integrated as a supportive adjunct—particularly in settings with limited specialist resources—to improve diagnostic reproducibility and streamline clinical workflows.

The deployment of AI-assisted tools in clinical settings comes with key challenges, including the need for thorough validation across diverse patient populations, adherence to patient data protection standards, and clear assignment of medico-legal responsibility. Since liability for medical errors associated with AI remains unclear, clinicians should ensure that any use of AI aligns with the recognized standard of care. Future research should focus on multicenter trials, development of models that integrate imaging with clinical data, and comprehensive cost-effectiveness analyses to ensure the practical applicability of AI-assisted methods in the clinical workflow. The successful integration of AI instruments, designed to complement human judgement instead of replacing it, may ultimately redefine SpA management, providing optimal and comprehensive patient care.
